# Leu124Serfs*26, a novel *AGPAT2* mutation in congenital generalized lipodystrophy with early cardiovascular complications

**DOI:** 10.1186/s13098-020-00538-y

**Published:** 2020-04-06

**Authors:** Renan Magalhães Montenegro Junior, Grayce Ellen da Cruz Paiva Lima, Virgínia Oliveira Fernandes, Ana Paula Dias Rangel Montenegro, Clarisse Mourão Melo Ponte, Lívia Vasconcelos Martins, Daniel Pascoalino Pinheiro, Maria Elisabete Amaral de Moraes, Manoel Odorico de Moraes Filho, Catarina Brasil d’Alva

**Affiliations:** 1 Brazilian Group for the Study of Inherited and Acquired Lipodystrophies (BRAZLIPO), Faculdade de Medicina, Universidade Federal do Ceará, Rua Professor Costa Mendes, 1608, Rodolfo Teófilo, Fortaleza, Ceará 60416200 Brazil; 2 Drug Research and Development Center—NPDM, Universidade Federal do Ceará, Fortaleza, Brazil

**Keywords:** Congenital generalized lipodystrophy, *AGPAT2*, Cardiovascular disease

## Abstract

**Background:**

Congenital generalized lipodystrophy (CGL) is a rare autosomal recessive disorder characterized by the near-total loss of subcutaneous adipose tissue soon after birth, resulting in ectopic fat deposition and severe metabolic disturbances. Most cases are caused by *AGPAT2* or *BSCL2* gene mutations. We aimed to report two unrelated CGL patients with a novel frameshift mutation in *AGPAT2* (p.Leu124Serfs*26).

**Methods:**

Clinical features and laboratory were obtained by medical interview and medical records review. DNA was extracted, amplified and sequenced. Mutation Taster was used to estimate the potential biological impact of the *AGPAT2* mutations on the protein function.

**Results:**

Patient 1: a 30-year-old woman with lipodystrophy phenotype at birth and diagnosis of diabetes at age 13 presented with severe hypertriglyceridemia and pancreatitis at age 17, hypertension and albuminuria at age 18, proliferative diabetic retinopathy with visual loss at age 25, and an acute myocardial infarction due to multivessel coronary disease during a hospitalization for forefoot amputation at age 29. At this time, she required hemodialysis due to end-stage renal disease. Patient 2: a 12-year-old girl with lipodystrophy phenotype and hypertriglyceridemia detected in the first year of life and abnormalities in the global longitudinal strain, evaluated by speckle-tracking echocardiography last year. Molecular analysis identified a c.369_372delGCTC (p.Leu124Serfs*26) *AGPAT2* mutation in both unrelated patients, a compound heterozygous mutation in Patient 1, and homozygous mutation in Patient 2.

**Conclusion:**

We describe two unrelated patients with type 1 CGL due to Leu124Serfs*26, a novel *AGPAT2* frameshift mutation, presenting as early cardiovascular disease. These findings suggest an association between Leu124Serfs*26 and a more aggressive phenotype.

## Background

Congenital generalized lipodystrophy (CGL; OMIN 608594) is a rare autosomal recessive disorder characterized by the total or near-total loss of subcutaneous adipose tissue soon after birth, resulting in ectopic fat deposition and metabolic complications. These patients present severe insulin resistance (IR), diabetes mellitus, hypertriglyceridemia, hepatic steatosis, and cardiovascular disease [1–3].

CGL is classified into four subtypes—1, 2, 3, and 4—according to the affected gene, *AGPAT2, BSCL2, CAV*-*1,* and *CAVIN1*, respectively. Most cases (95%) are subtypes 1 (CGL1) or 2 (CGL2). *AGPAT2* is located in chromosome 9q34 and encompasses six exons [4]. This gene codes a homonymous protein (1-acylglycerol-3-phosphate acyltransferase-β) that triggers the synthesis of triglycerides inside the adipocyte, converting lysophosphatidic acid to phosphatidic acid [5, 6]. This mutation influences the peroxisome proliferator-activated receptor gamma (PPARγ), the main regulator of adipocyte differentiation [7–9].

Different *AGPAT2* mutations have been described worldwide. This gene is expressed in various tissues, including the gastrointestinal tract, heart, kidney, and liver, among others [10]. Descriptions of the clinical characteristics of patients carrying new mutations are important since they may improve our understanding of multiorgan diseases and adipose tissue physiology, and this understanding may enable us to speculate about genotype–phenotype associations.

In this study, we reported two unrelated CGL1 patients with a novel frameshift mutation in *AGPAT2*, NM_006412:p.Leu124Serfs*26.

## Methods

**Fig. 1:**
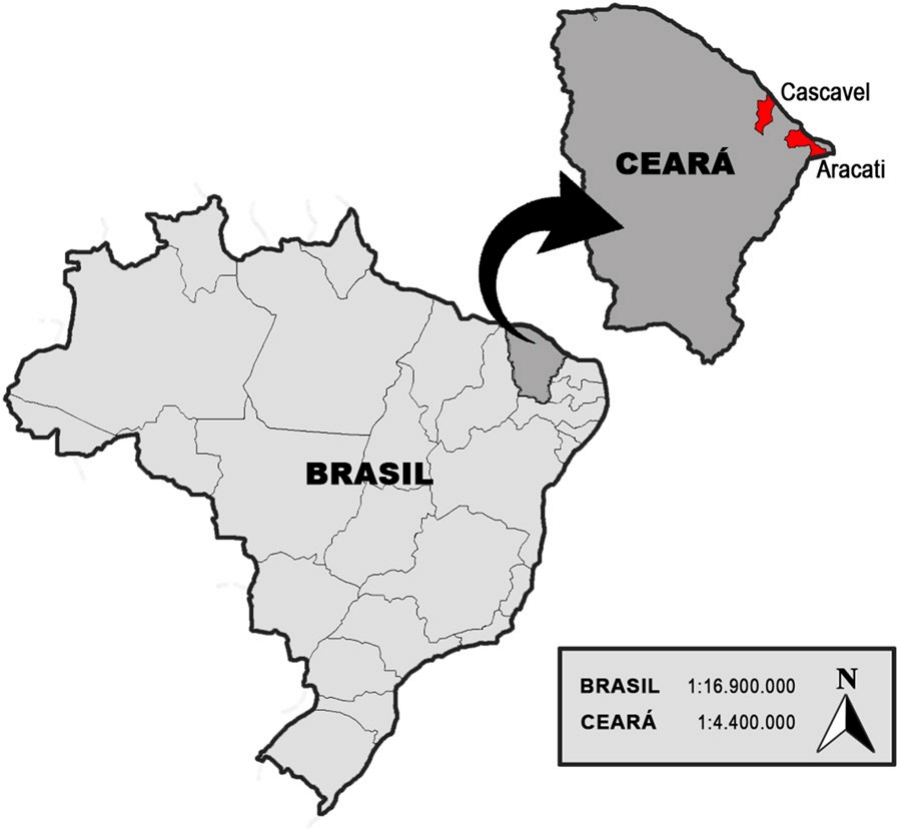
CGL pa–tients’ hometowns (in red) in the state of Ceará, Brazil

Clinical features and laboratory and molecular data from two Brazilian patients from the state of Ceará, Northeast Brazil (Fig. 1), were obtained by medical interview and medical records review.

Blood glucose, cholesterol, and triglyceride levels were obtained after overnight fast and determined according to standard methods using automated equipment. Glycohemoglobin A1c values were determined by ion exchange high-performance liquid chromatography (HPLC), and serum insulin levels were measured by immunoassays with reagents provided by Roche Diagnostics (Basel, Switzerland). Thyroid stimulating hormone (TSH) and thyroid hormones (fT4 and T3) were measured using chemiluminescence (ADVIA Centaur^®^ XP, Siemens). Plasma leptin levels were measured by an immunoassay using a commercial kit (DIAsource ImmunoAssays, Louvain-la-Neuve, Belgium). HOMA–IR (homeostasis model assessment) was also calculated (fasting glycaemia (mmol/L) × fasting insulin (μU/mL))÷22.5) [11].

Standard in-house protocols based on a salting-out method were used to extract genomic DNA from peripheral blood leucocytes [12]. The entire coding regions and intron–exon junctions of *AGPAT2* and *BSCL2* were amplified by polymerase chain reactions (PCR) using specific primers and were automatically sequenced; primer sequences are listed in Table 1. Amplification reactions were executed in a final volume of 25 µL, comprising 200 ng genomic DNA, 0.2 mM dNTPs, 1.5 mM PCR_x_ Enhancer Solution (Invitrogen), 0.6 pmol of each primer, 1× PCR buffer, and 1U GoTaq^®^ DNA polymerase (Promega, Madison, WI), and were executed for 35 cycles: denaturation at 95 °C for 30 s, annealing at 55–62 °C for 30 s, extension at 72 °C for 1 min, and a final extension for 10 min at 72 °C. The PCR products were checked on 1% agarose gel electrophoresis, purified, and automatically sequenced in an ABI PRISM™ 3100 Genetic Analyzer automatic DNA sequencer (Applied Biosystems, Foster City, CA). All sequence variations were assessed on both strands and repeated in a second PCR.

**Table 1:** Oligonucleotides used for *AGPAT2* amplification and sequencing

**Primer**	
AGPAT2-1F AGPAT2-1R	5′-cgcaataaggggcctgag-3′ 5′-ggaccccctcctgtgc-3′
AGPAT2-2F AGPAT2-2R	5′-gggactctgtccgcttca-3′ 5′-cagccctgtgtcctcgtc-3′
AGPAT2-3F AGPAT2-3R	5′-ggtgctcagcagctgtcttc-3′ 5′-tttctgccaaaaccaagtcac-3′
AGPAT2-4F AGPAT2-4R	5′-aaaacaagacccccacatcat-3′ 5′-gaggagtcccttgtgtgtcaag-3′
AGPAT2-5F AGPAT2-5R	5′-cctcagctgtgcgtctcc-3′ 5′-gagtcactcattcgccacat-3′
AGPAT2-6F AGPAT2-6R	5′-ctagggagtccaggggaaga-3′ 5′-agtgacagaaggggcttcct-3′

Mutation Taster was used to estimate the potential biological impact of the *AGPAT2* mutations on the protein function. This is a web-based application that performs a battery of in silico tests on protein and DNA level to estimate the impact of a variant on the gene product. It predicts the functional consequences of amino acid substitutions as well as intronic and synonymous alterations, short insertion and deletion mutations, and variants spanning intro-exon borders. The program filter harmless mutations from the disease-causing ones by comparison with integrated databases and perform tests to determine the nature of the given variant, that comprises the amino acid substitutions, conservation of affected amino acids, potential loss of function protein domains, length of protein, conservation on DNA level, among others. The result is assessed by a probabilistic classifier which decides if the combined effect of the variant might be deleterious for the protein. The test output explains if this is a predicted pathogenical mutation with high accuracy and gives detailed information about it [13].

## Results

### Study subjects

#### Patient 1

**Fig. 2:**
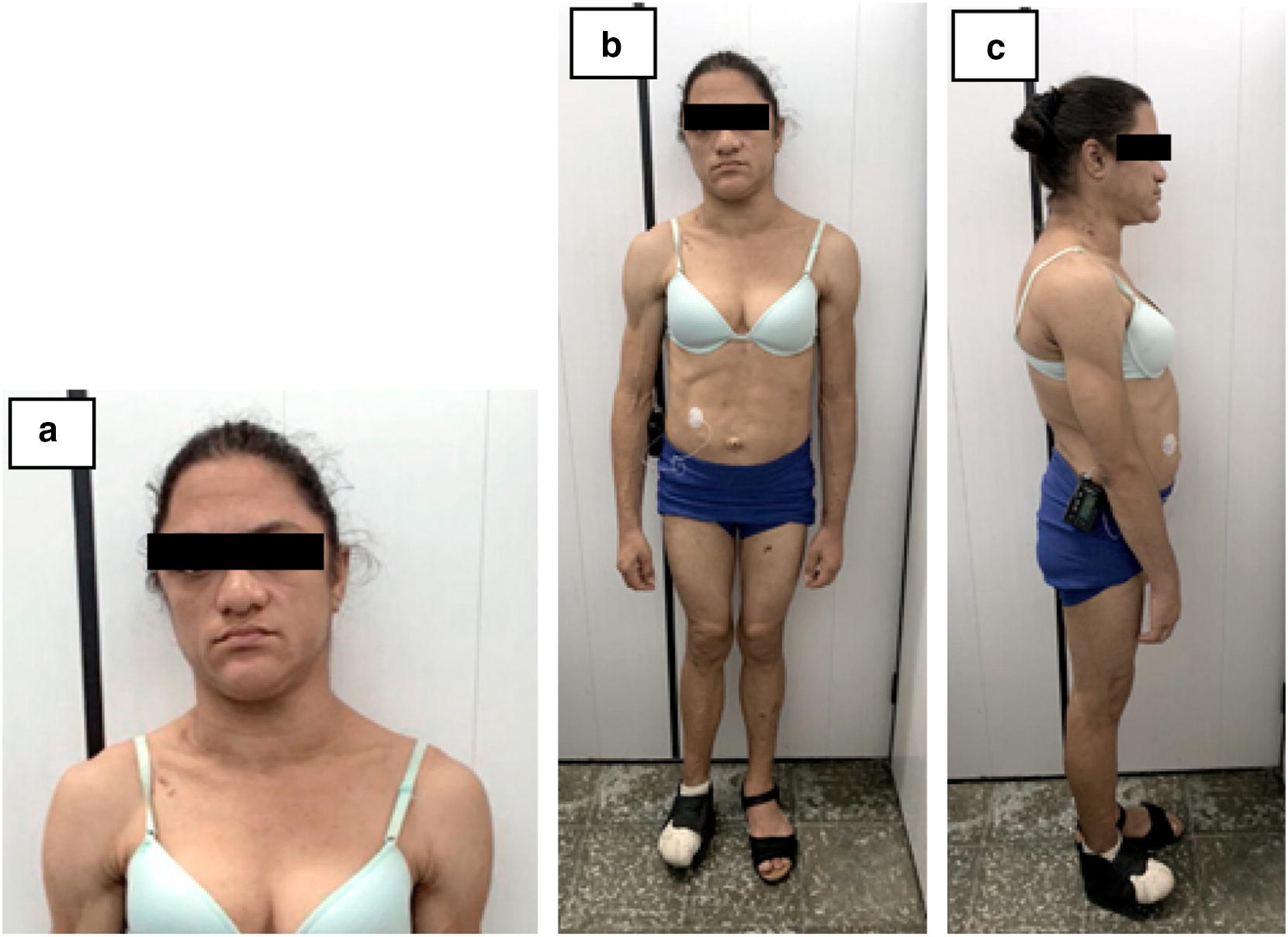
Patient 1, a 30-year-old woman with type 1 congenital generalized lipodystrophy. **a** acromegaloid facies; **b**, **c** generalized lack of subcutaneous fat, muscular hyperplasia, umbilical hernia, and increased abdominal volume

Patient 1 was a 30-year-old woman (Fig. 2), non-smoker, born full-term (unknown birth weight) as the third child of nonconsanguineous parents in the city of Cascavel, state of Ceará, Brazil (Fig. 1). Lipodystrophic phenotype was detected at birth and the diagnosis of CGL was given at the age of 6 months when she was hospitalized due to a respiratory infection. Hypertriglyceridemia (223 mg/dL) was also detected at that time.

She had no clinical follow-up until the age 13, when she was diagnosed with diabetes mellitus and, 2 years later, she was referred to our institution with poor glycemic control (fasting bood glucose 266 mg/dL) and hypertriglyceridemia (816 mg/dL). At that time, her insulin dose was adjusted from 2.1 to 2.3UI/Kg/day and she was started on metformin 1700 mg/day, and ciprofibrate 100 mg/day. However, she maintained poor metabolic control (A1c 12%), with inadequate dietary adherence, and presented two episodes of pancreatitis related to hypertriglyceridemia (2386 mg/dL) at age 17. One year later, she was diagnosed with hypertension and diabetic nephropathy (albuminuria) and was started on enalapril.

At age 20, she was diagnosed with subclinical primary hyperthyroidism (TSH 0144 uU/mL and free T4 1,1 ng/dL) with a diffuse goiter, fourfold increase, with six small benign nodules on cytology (Bethesda 2 category), despite having lived in an iodine-sufficient region. She was started on methimazole.

At age 25, she presented with proliferative diabetic retinopathy and visual loss in the right eye, peripheral neuropathy, and cardiac autonomic dysfunction manifested by dizziness, resting tachycardia, and orthostatic hypotension (Table 2).

**Table 2:** Laboratory tests, abdominal ultrasound, and echocardiography of congenital generalized lipodystrophy Patients 1 and 2

Patient	1		2	
Age (years old)	15*	30**	1*	12.2**
Leptin (ng/mL)*	1.0	–	1.0	–
Total cholesterol (mg/dL)	292	175	131	145
HDL (mg/dL)	–	25	26	18
HOMA-IR	NA	NA	1.8	9.9
Glucose (fasting) (mg/dL)	259	291	82	75
GlycohemoglobinA1c (%)	–	6.4	5.1	5.7
Triglycerides (mg/dL)	816	306	519	397
Abdominal ultrasound	–	Renal microlithiasis, hepatomegaly, hepatic steatosis, and nephromegaly	–	Hepatomegaly, hepatic steatosis, and nephromegaly
Cardiovascular autonomic neuropathy tests^a^	–	Clinical/ Advanced	–	Absent
Conventional echocardiography	–	increased left atrium, apical hypokinesia, preserved systolic function	–	Normal
GLS by speckle-tracking echocardiography	–	− 17.9%^b^	–	− 19.3%^b^

At the first * and the last ** clinical evaluation

^a^Cardiovascular autonomic neuropathy tests: deep breathing test (E/I coefficient), valsalva maneuver, orthostatic test (30/15 coefficient) and orthostatic or postural hypotension test

^b^GLS: Global longitudinal strain, reference value (adults): − 21.1 to − 19.4; (2–9 years old): − 23.9 to − 22

At age 29, she presented a diabetic foot ulcer that had progressed to necrotizing fasciitis and severe sepsis, leading to a forefoot amputation. During that same hospitalization, she had an acute myocardial infarction and underwent the placement of three coronary drug-eluting stents due to multivessel coronary artery disease. The two-dimensional speckle-tracking echocardiography showed moderate increase of the left atrium, left ventricular concentric hypertrophy, and left ventricular dysfunction.

At age 29, her diabetic nephropathy progressed to end-stage renal disease and she was started on hemodialysis.

She experienced menarche at age 17 and had two spontaneous pregnancies (at ages 22 and 26), despite a diagnosis of polycystic ovarian syndrome. Her first pregnancy resulted in a spontaneous fetal loss in the first trimester. The second pregnancy had a favorable outcome, with a healthy offspring with no CGL phenotype. No similar cases have been reported in her family.

At the time of examination, Patient 1 was 160.5 cm tall, weighed 58.9 kg, and had a BMI of 22.9 kg/m^2^. She had acromegaloid facies and a generalized lack of subcutaneous fat, with preserved fat in the palmar and plantar regions, intense acanthosis nigricans (cervical, axillary, and inguinal region), extreme muscularity, phlebomegaly, umbilical hernia, and hepatomegaly. She had no signs of intellectual impairment. Table 2 shows the main laboratory and complementary exams performed during her follow-up.

#### Patient 2

**Fig. 3:**
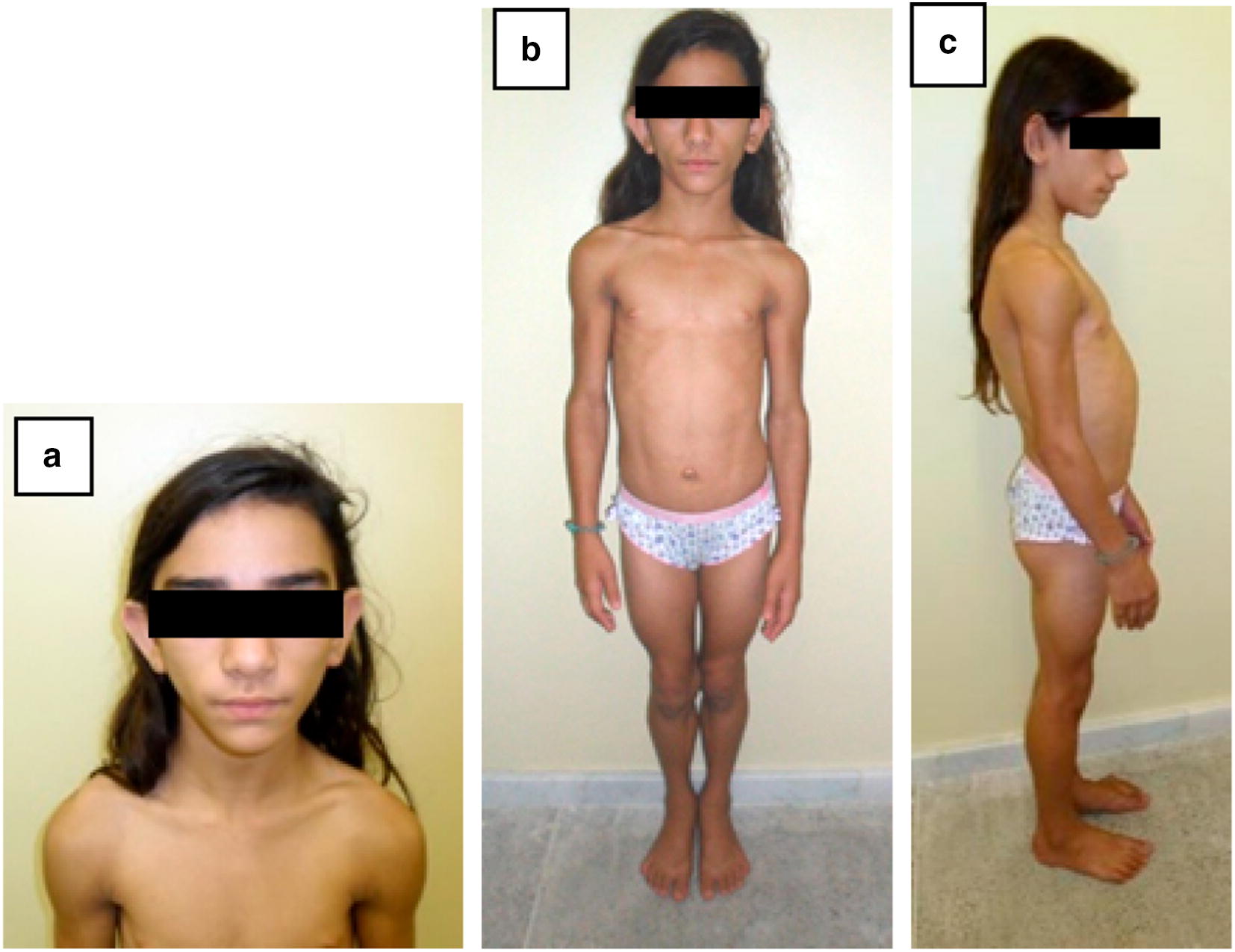
Patient 2, a 12-year-old girl with type 1 congenital generalized lipodystrophy. **a** acromegaloid facies; **b**, **c** generalized lack of subcutaneous fat, muscular hyperplasia, umbilical hernia, and increased abdominal volume

Patient 2 was a 12-year-old girl (Fig. 3), non-smoker, born full-term (birth weight: 3570 g; length: 49 cm) as an only child of consanguineous parents (second cousins) in the city of Aracati, state of Ceará, Brazil (Fig. 1). Lipodystrophic phenotype was detected at the age of 5 months.

At age 1, hypertriglyceridemia (519 mg/dL), acanthosis nigricans, and hepatomegaly were detected. She had thelarche at age 6.5 and pubarche at age 6.7 and, although she had not been treated, she had not yet presented with menarche. She was adherent to non-drug therapy during her follow up. At age 12, she was started on metformin due to pre-diabetes (A1c: 5.7%). No similar cases have been reported in her family.

At the time of physical examination, she was 163 cm tall (Z: +1.0), weighed 49.3 kg, and her BMI was 18.6 kg/m^2^ (SD: 0.08). Her pubertal status was M4/P5 (Tanner Stage); hirsutism was present. She had acromegalic facies and a generalized lack of subcutaneous fat, with preserved fat in the palmar and plantar regions, intense acanthosis nigricans, extreme muscularity, umbilical hernia, and hepatomegaly. She had no signs of intellectual impairment. Table 2 shows the main laboratory and complementary exams performed during her follow-up.

### Molecular results

Molecular analysis of Patient 1 identified two *AGPAT2* mutations in heterozygosis configuring a compound heterozygous. The first mutation was a deletion of nucleotides GCTC at positions 369–372, located in exon 3 (NM_006412:c.369_372delGCTC, p.Leu124Serfs*26). The second mutation was the previously described A-for-G substitution at position -2 of the intron 4 (c.589–2A > G).

**Fig. 4:**
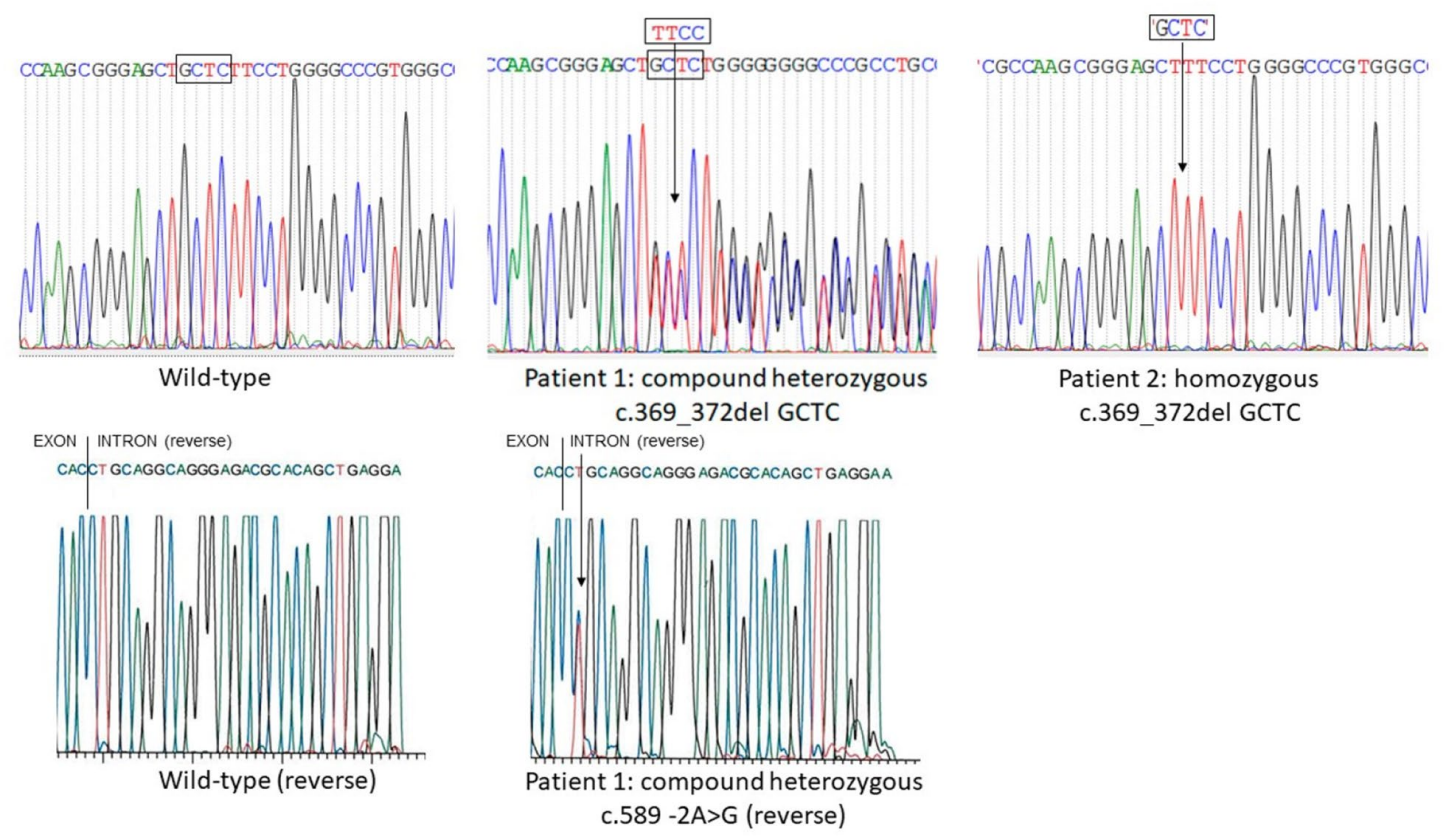
Electropherogram of exon 3 showing the wild-type (**a**) and the novel *AGPAT2* mutation (c.369_372del GCTC) in the congenital generalized lipodystrophy Patients 1 (**b**) and 2 (**c**). Electropherogram of intron4/exon 5 showing the wild-type (**d**) and the already described *AGPAT2* mutation (c.589 -2A > G) in the congenital generalized lipodystrophy Patient 1 (**e**)

Patient 2 harbored the deletion of the GCTC nucleotides at positions 369–372 of the transcript, located in exon 3 (NM_006412:c.369_372delGCTC) in a homozygous state (Fig. 4).

The potential impact of the p.Leu124Serfs*26 mutation on 1-AGPAT2 protein function was evaluated by the Mutation Taster tool, being considered disease causing. The probability value, i.e. the probability of prediction was 1, meaning a “high security” of the disease causing prediction.

## Discussion

In this paper, we have described in detail a novel pathogenic mutation in *AGPAT2*, Leu124Serfs*26; and investigated its potential impact on the clinical features of CGL in two unrelated patients.

Molecular analysis identified a deletion of 4 nucleotides at position 369–372 of the exon 3, resulting in substitution of a leucine (hydrophobic amino acid) to serine (neutral polar amino acid) at codon 124 and a stop codon at the 26th position from the first amino acid changed, generating a truncated protein. Some in silico tools can be used to assess possible effects of an amino-acid substitution on the function and structure of human proteins, using straightforward physical and comparative considerations. Leu124Serfs*26 was analyzed using Mutation Taster tool and was predicted to be pathogenic, with a high probability value. It generated a truncated protein, probably causing a drastic alteration in the protein function, which is related to the CGL1 phenotype. A previous article on cardiovascular autonomic neuropathy in our sample of CGL patients mentioned this mutation in a supplementary table. However, it did not describe the clinical characteristics of these two patients [14].

The 1-AGPAT2 is a membrane bound protein located in the endoplasmic reticulum that consists of 278 amino acids with two highly conserved motifs among the other acyltransferases; NHX_4_D (amino acids 97–103) and EGTR (amino acids 172–175) are both crucial for enzymatic activity [15, 16]. The NHX4D motif is cytoplasmically oriented whereas the EGTR motif is in the lumen of the endoplasmic reticulum. To be catalytically active, both motifs should come together on the same side of the membrane [10]. The Leu124Serfs*26 mutation affects the second highly conserved motif, making a complete loss of enzymatic activity predictable.

**Fig. 5:**
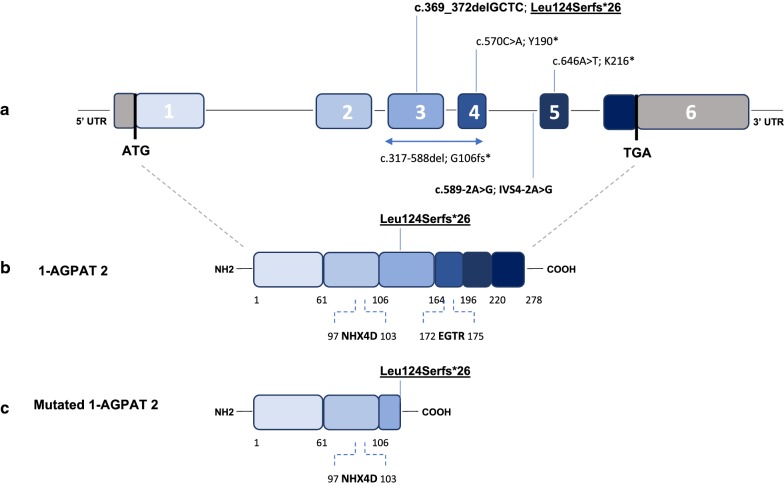
**a** Genomic map of the AGPAT2 gene showing the main mutations reported in Brazilian patients with lipodystrophy. Mutations in bold are those reported in this study. The underlined mutation is the novel AGPAT2 frameshift mutation reported in this study. Numbered boxes represent exons and the in-between lines indicate introns. **b** Schematic of the 1-AGPAT2 protein, showing the two conserved motifs, NHX4D and EGTR, and localizing the novel mutation reported in this study. **c** Schematic of the mutated 1-AGPAT2 protein resulting from the Leu124Serfs*26 mutation which leads to a stop codon at the 26th position from the first amino acid changed, generating a truncated protein that misses the EGTR motif

Patient 1 also carried the c.589-2A > G mutation in her *AGPAT2* gene, characterizing a compound heterozygous with the c.369_372delGCTC, p.Leu124Serfs*26 mutation. Thirty-seven different mutations in *AGPAT2* have been described worldwide related to congenital generalized lipodystrophy [17]. Globally, the most common mutation is the c.589-2A > G (rs116807569). In Brazil, the predominant CGL mutations are c.589-2A > G, c.317-588del, c.646A > T, and c.570C > A in the AGPAT2 gene [17–19]. Our historical relationship with Portugal and Africa during colonization and the previous description of the c.589-2A > G mutation in these locations suggest a founder effect (Fig. 5) [17, 20].

Both patients manifested early metabolic complications. Patient 1 had two episodes of pancreatitis related to hypertriglyceridemia and a diagnosis of diabetes at age 13 with poor glycemic control. Patient 2 also presented with hypertriglyceridemia (in the first year of life) and had pre-diabetes detected at age 12, despite having adherence to the non-drug therapy. According to the literature, about half of CGL patients have the onset of diabetes at puberty [7]. Agarwal et al. [20] reported 18/38 CGL1 patients with diabetes, with the mean age of diabetes onset at age 16.8 ± 10.1, and 8/16 CGL2 patients with diabetes who had an earlier diabetes onset at the mean age of 9.5 ± 3.1 years. In a large Brazilian series, where approximately 70% had CGL2, the mean age of diabetes onset was 15.8 ± 7.1 years old [19].

Patient 1 had severe and early micro- and macrovascular complications detected approximately 5 years after the diabetes diagnosis, as well as end-stage renal disease, autonomic cardiovascular neuropathy and acute myocardial infarction at age 29. Ponte et al. [14] observed early commitment of cardiovascular autonomic modulation in 10/20 young CGL patients, detected through abnormalities in cardiovascular autonomic neuropathy tests.

High rates of coronary artery disease (CAD) would be expected to be more common in CGL patients due to the severity of metabolic complications. Otherwise, the CAD complications are not usually described in patients with CGL [19, 21–23]. Few cases of CAD have been reported [21]. Agarwal et al. [20] reported that 13% of 38 CGL1 patients had cardiomegaly but did not observe any cases of CAD. Lupsa et al. [22] observed 1/19 CGL1 patients with coronary artery disease; the one was a woman (unknown mutation) who underwent myocardial revascularization at age 45. In a Turkish series, 3/16 CGL1 patients had CAD at age 30, 62, and 62, respectively, carrying c.662–2A > C (IVS5–2A > C), c.202C > T (p.R68*), and c.685G > T (p.E229*) *AGPAT2* mutations. The younger one had also renal failure [23].

Patient 2 had already presented abnormalities in the global longitudinal strain, evaluated by speckle-tracking echocardiography (2D-STE), at an incredibly young age. The 2D-STE is a promising echocardiography technique that plays an important role in early detection of cardiac dysfunction. It is more sensitive than ejection fraction to detect subclinical left ventricular systolic dysfunction, because evaluates percentage of myocardial fiber deformation throughout the cardiac cycle [24]. A recent study described that 15/22 (68.2%) young patients with CGL had left ventricular systolic dysfunction detected by 2D-STE, even with normal conventional echocardiography [25].

Peripheral arterial disease is a common macrovascular complication in diabetic patients but is rarely described in CGL patients. In a Turkish series, lower limb amputations were described in 2/16 patients at ages 25 and 30, carrying c.646A > T (p.K216*) and c.662–2A > C (IVS5–2A > C) *AGPAT2* mutations, respectively [23]. Our Patient 1 presented with a foot ulcer, evolving with forefoot amputation and characterizing a severe and precocious vascular complication.

Patient 1 presented with asymptomatic renal lithiasis. The most common renal finding in CGL is nephromegaly, but renal lithiasis was also described in a 2-month-old Persian child who carried a nonsense mutation in exon 6 of *AGPAT2* (c.685G > T, p.Glu229*) [26]. Obesity, diabetes, and metabolic syndrome have been associated with renal lithiasis [27, 28]. Insulin resistance inhibits proximal tubule ammoniagenesis, causing an acidic milieu that increases the risk of uric acid calculi, but this has been related to cases with high body-mass indexes [29]. Another reason for the presence of urolithiasis could be the high intake of animal proteins and polyphagia in obese people, which is also observed in CGL patients due to leptin deficiency [30].

The thyroid can be affected by IR through the anabolic action of high insulin levels. The clinical manifestations are larger thyroid volume and the genesis of nodules [31–33]. Patient 1 had a diffuse goiter along with cold nodules. There are few descriptions of thyroid abnormalities in patients with CGL. Akinci et al. [23] described a female 62-year-old patient with toxic multinodular goiter who was a carrier of the c.202C > T (p.R68*) mutation.

Patient 1 presented with late menarche, and Patient 2 exhibited precocious puberty (thelarche, pubarche, increased growth velocity), but, even untreated, had still not experienced menarche. Although patients with CGL have accelerated growth [7] and precocious thelarche and/or pubarche (unpublished data), primary or secondary amenorrhea are common [34, 35]. High levels of androgens related to insulin resistance may contribute to the precocious pubarche [5, 35].

Pregnancy is rare in CGL, and only two patients have been reported with a favorable outcome [36]. Loss of the pulsatile secretion of gonadotropins and hyperandrogenism may impair ovulation and pregnancy, and poorly controlled diabetes in CGL patients gives rise to high-risk pregnancies [7]. However, our Patient 1 had one pregnancy with a favorable outcome and a healthy offspring with no CGL phenotype, despite Patient 1’s poor glycemic control.

This study had some limitations. Genetic information of the patients’ ancestors was not available, and the possibility of a founder effect was not evaluated. Additionally, it is not possible to be sure of a genotype–phenotype correlation based only on two case reports.

## Conclusion

We described two unrelated patients with type 1 congenital generalized lipodystrophy born in the same Brazilian region presenting with the Leu124Serfs*26, a novel frameshift mutation in *AGPAT2*. Their clinical manifestations were characterized by early and severe cardiovascular disease, which suggests a possible association between this mutation and a more aggressive phenotype. However, additional studies are needed, as a cell culture of a homozygous patient, to better stablish the role of the new discovered mutation.

## Data Availability

The datasets generated during and/or analyzed during the current study are available from the corresponding author upon reasonable request.

## Abbreviations

A1c: Glycohemoglobin A1c
*AGPAT2*: 1-Acylglycerol-3-phosphate O-acyltransferase 2 gene
1-AGPAT2: 1-Acylglycerol-3-phosphate O-acyltransferase 2 protein
BRAZLIPO: Brazilian Group for the Study of Inherited and Acquired Lipodystrophies
*BSCL2*: Bernardinelli–Seip congenital lipodystrophy type 2 gene
BSCL2: Bernardinelli–Seip congenital lipodystrophy type 2 protein
CAD: Coronary artery disease
*CAVIN1*: Caveolae associate protein 1
*CAV*-*1*: CAVEOLIN 1 gene
CGL: Congenital generalized lipodystrophy
CGL1: Congenital generalized lipodystrophy type 1
CGL2: Congenital generalized lipodystrophy type 2
DNA: Deoxyribonucleic acid
DM: Diabetes mellitus
fT4: Free tiroxine
GLS: Global longitudinal strain
HOMA-IR: Homeostasis model assessment of insulin resistance
HPLC: High-performance liquid chromatography
IR: Insulin resistance
M4/P5: Tanner stage
OMIM: Online Mendelian inheritance in man
PCR: Polymerase chain reaction
PPARγ: Peroxisome proliferator-activated receptor gamma
SD: Standard deviation
TSH: Thyroid stimulating hormone
T3: Triiodothyronine
